# PhiC31 recombination system demonstrates heritable germinal transmission of site-specific excision from the *Arabidopsis *genome

**DOI:** 10.1186/1472-6750-10-17

**Published:** 2010-02-23

**Authors:** James G Thomson, Ronald Chan, Roger Thilmony, Yuan-Yeu Yau, David W Ow

**Affiliations:** 1 Crop Improvement and Utilization Research Unit, Western Regional Research Center, USDA-ARS, 800 Buchanan Street, Albany CA, 94710, USA; 2 Plant Gene Expression Center and UC Berkeley, 800 Buchanan Street, Albany CA, 94710, USA; 3 Current address: South China Botanical Garden, Xingke Road 723, Tianhe, Guangzhou, China 510650

## Abstract

**Background:**

The large serine recombinase phiC31 from broad host range *Streptomyces *temperate phage, catalyzes the site-specific recombination of two recognition sites that differ in sequence, typically known as attachment sites *attB *and *attP*. Previously, we characterized the phiC31 catalytic activity and modes of action in the fission yeast *Schizosaccharomyces pombe*.

**Results:**

In this work, the *phiC31 *recombinase gene was placed under the control of the *Arabidopsis OXS3 *promoter and introduced into *Arabidopsis *harboring a chromosomally integrated *attB *and *attP*-flanked target sequence. The phiC31 recombinase excised the *attB *and *attP*-flanked DNA, and the excision event was detected in subsequent generations in the absence of the *phiC31 *gene, indicating germinal transmission was possible. We further verified that the genomic excision was conservative and that introduction of a functional recombinase can be achieved through secondary transformation as well as manual crossing.

**Conclusion:**

The phiC31 system performs site-specific recombination in germinal tissue, a prerequisite for generating stable lines with unwanted DNA removed. The precise site-specific deletion by phiC31 *in planta *demonstrates that the recombinase can be used to remove selectable markers or other introduced transgenes that are no longer desired and therefore can be a useful tool for genome engineering in plants.

## Background

Plant biotechnology has a role in addressing global needs for food, fiber and fuel, by developing new crop varieties with increased pest resistance, biofortification, and abiotic stress tolerance. Publicly acceptable forms of biotechnology offer an avenue for meeting these demands [1]. Recombinase-mediated genetic engineering provides a favorable direction for enhancing the precision of biotechnological approaches. Concerns over the presence of antibiotic resistance genes in the food supply and their escape into the environment [2] can be relieved through the use of recombinase technology to excise unwanted DNA from the genome of genetically engineered (GE) crops prior to marketing or release [3,4]. A study by Chawla and colleagues [5] documented how site-specific integration in rice exhibited stable gene expression over multiple generations. The research also demonstrated that rice with multicopy transgene inserts, initially silenced for expression, recovered expression when resolved by recombinase technology to a single genomic copy. Such studies demonstrate other potential uses for recombinase technology in the development of plant biotechnology.

Genomic engineering took a large step forward with the discovery that site-specific recombinases, a group of enzymes that are capable of precise DNA cleavage and ligation without the gain or loss of nucleotides, could facilitate conservative DNA manipulation in a heterologous host [6]. The recombinase super family is split into two fundamental groups, the tyrosine and serine enzymes. This grouping is based on the active amino acid (Y or S) within the catalytic domain of each enzyme family. The best known tyrosine recombinases are Cre, Flp and R [7]. Tyrosine recombinases utilize identical recognition sites and perform a bi-directional mode of recombination. They have been shown to be effective for excision of unwanted DNA from the genome of the host but require complex schemes for integration.

The serine enzyme group includes the phiC31, TP901-1 and Bxb1 recombinases among others [8,9]. Members of this group recognize two non-identical recognition sites (*attB *and *attP*) and perform a uni-directional mode of recombination. While less research has been conducted on this group, it appears that the serine enzymes are well suited for precise genomic recombination due to their uni-directional catalytic activity that prevents the reversion of recombination products.

In previous studies, we identified a number of prokaryotic site-specific recombination systems that function in the eukaryote *Schizosaccharomyces pombe *[8,10]. Among those, the phiC31 uni-directional recombinase was highly efficient. The system has been successfully shown capable of recombinase mediated excision, inversion and integration reactions. The phiC31-*att *system is derived from the broad host range *Streptomyces *temperate phage phiC31 [11]. The 613 amino acid phiC31 protein acts on recognition sites *attB *and *attP *that are minimally 34 bp and 39 bp, respectively [12]. Published evidence has demonstrated that the phiC31 system is functional for excision and transmission of marker-free plastids in the seed of tobacco and in the genome of *Arabidopsis *and wheat [13-17] but has yet to be demonstrated capable of germinal transmission of nuclear DNA *in planta*.

In this research, we tested the phiC31 recombination system for the capacity to germinally transmit a target sequence that has undergone site-specific excision from within the *Arabidopsis *genome to a subsequent generation in the absence of the recombinase gene. Plants transgenic for an *attB *and *attP *flanked target sequence were introduced with a second construct that contained the recombinase gene. The phiC31 recombinase performed excision of the target sequence from three independent plant lines (i.e. genomic locations) and generated stably excised progeny plants that carry only the recombined target DNA of interest in the absence of the recombinase gene. This demonstrates that the phiC31 recombination system is suitable for the generation of stable marker-free, recombinase-free transgenic plants.

## Results

### Experimental design

**Figure 1:**
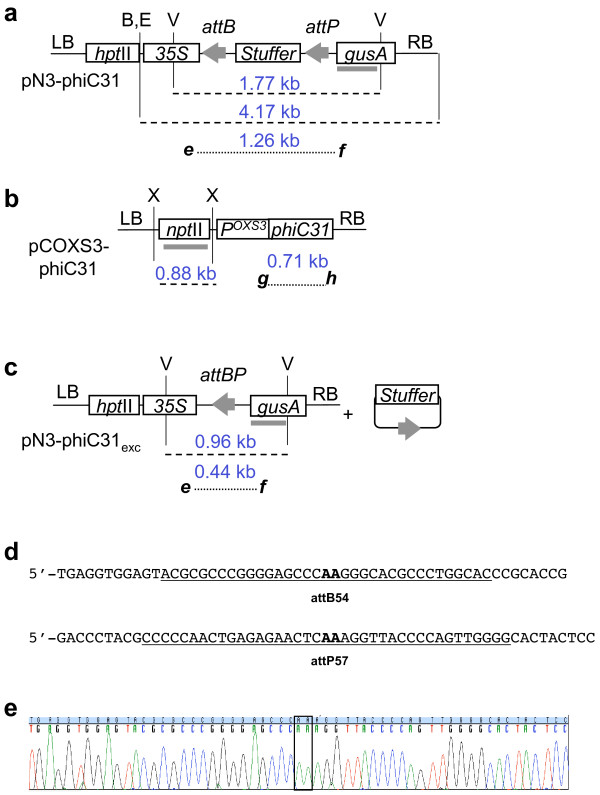
**T-DNA structures**. (not to scale) from a) pN3-phiC31; b) pCOXS3-phiC31; and c) predicted single copy T-DNA structures after excision of stuffer by phiC31-*att *recombination. PCR primers shown as ***e***, ***f***, ***g***, ***h***; *att *sites as grey arrowheads; hybridization probes as grey rectangles. Abbreviations: B, *Bam*HI; E, *Eco*RI; V, *Eco*RV; X, *Xho*I; RB, T-DNA right border; LB, T-DNA left border. Length in kb of PCR products (dotted lines) and DNA fragments (dashed lines). d) Sequence of the 54 bp *attB *and 57 bp *attP *phiC31 recognition sites, where the minimal required sequence is underlined and the 2 nucleotide '**AA**' core region of crossover is in bold. e) sequence of a PCR product detecting a conservative site-specific excision event. Not shown are gene terminators and promoters for *hpt*II (*hygromycin phosphotransferase *II) and *npt*II (*neomycin phosphotransferase *II) and the gene terminator for *gusA *(*β*-*glucuronidase*).

**Figure 2:**
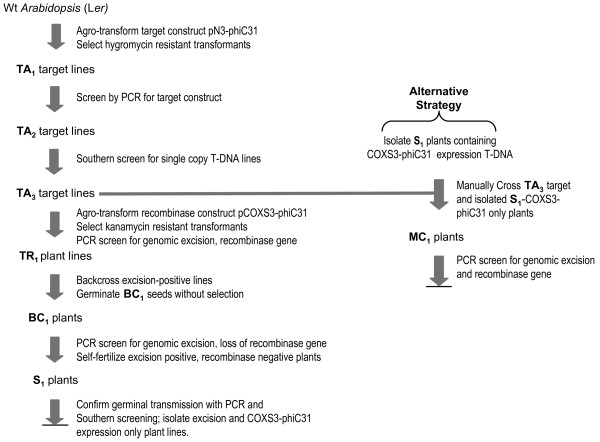
**Strategy for generating site-specific excision plant lines**.

To test for site-specific recombination, we initially sought to use a gain-of-function strategy whereby excision of a transgene would lead to promoter fusion with a previously distal marker [18]. Hence, pN3-phiC31 was configured with a CaMV 35S promoter (35S) proximal to a 760 bp non-coding stuffer region followed by a distal *gusA *coding region (Fig. 1a). The stuffer region is flanked in direct orientation by the 54 bp *attB *and 57 bp *attP *phiC31 attachment sites (Fig. 1d) derived from pPB-phiC31 [8] located in the binary vector pCambia 1301 http://www.cambia.org/daisy/cambia. The expectation was that prior to site-specific recombination, 35S would not drive expression of *gusA *due the presence of the stuffer region. After recombination, the non-coding stuffer would be removed and activate expression of *gusA *(Fig. 1c). In this strategy, we first introduced the recombination target (pN3-phiC31) into the *Arabidopsis *genome via *Agrobacterium *transformation. The target construct contains *hpt*II (*hygromycin phosphotransferase *II) for selection of transgenic plants and was intentionally placed outside of the recombination recognition sites (and thus is not excised by phiC31) to aid the tracking of excised plants. These target lines, or 'TA' lines, were then transformed with the second construct, pCOXS3-phiC31 (Fig. 1b) that expresses the recombinase gene to produce the 'TR' lines. Upon site-specific excision of the recognition site-flanked DNA, the TR_1 _plants were backcrossed to wild type plants and the BC_1 _progeny screened for segregants that retain the excision event but lack the recombinase gene (Fig. 2).

### Target lines for phiC31 recombination

The target construct pN3-phiC31 was introduced into *Arabidopsis *and 23 hygromycin resistant lines were confirmed by PCR detection of a 1.26 kb product that spans the recognition site-flanked non-coding stuffer region (data not shown). Of those, 13 pN3-phiC31 lines were propagated to the TA_2 _generation and examined by Southern blot for single copy T-DNA integration. *Eco*RI or *Bam*HI each cuts once within the target T- DNA (Fig. 1a). Hybridization with a *gusA *probe of *Eco*RI or *Bam*HI cleaved genomic DNA should reveal a band size >4.17 kb, the length of the cleaved T-DNA. A hybridizing band <4.17 kb would indicate integration of a truncated T-DNA. From this analysis, three of the 13 pN3-phiC31 plants were determined to contain a single copy of a likely complete T-DNA (data not shown) and designated TA_2_-phiC31.22, 31, and 34. The 1.26 kb PCR product from each of these lines was sequenced to confirm the presence of intact *attB *and *attP *sites (Fig. 1d).

### Arabidopsis OXS3 promoter for expression of phiC31

As previous research has demonstrated successful germline tissue expression of the *parA *and *cre *recombinase genes [19], we chose the 1.5 kb promoter fragment of the *Arabidopsis ****Ox****idative ****S****tress ****3 ***gene (*OXS3*) (AGI At5g56550) for *phiC31 *gene expression and termed the plasmid pCOXS3-phiC31 (Fig. 1b). Independent research, through the use of tiling microarrays, has also confirmed that the *OXS3 *gene is constitutively expressed in most *Arabidopsis *tissues [20,21].

### Secondary transformation of TA target lines

**Figure 3:**
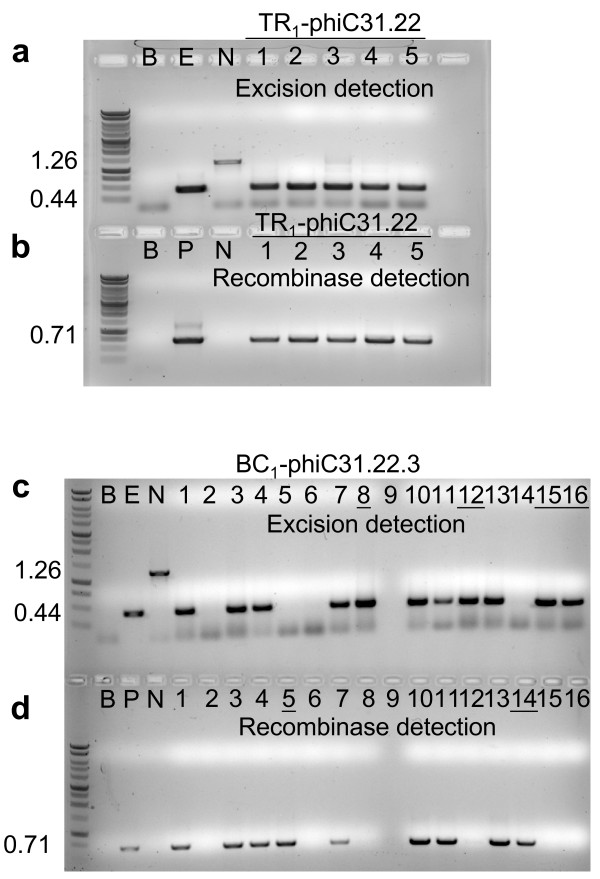
**PCR analysis for site-specific recombination and the presence of the *phiC31 *gene in the TR_1 _and BC_1 _generations**. PCR reactions (a, c) with primers ***e ***and ***f ***(**Fig. 1**) or (b, d) with primers ***g ***and ***h ***(**Fig. 1**) on representative plant DNAs. a, b) retransformed TR_1_- phiC31.22 lines. c, d) Back Crossed line BC_1_-phiC31.22.3. Unlined numbers represent excision or recombinase only plants lines. Control lanes are B (blank, no DNA); E (excision, pN3-phiC31_exc_); N (no excision, pN3-phiC31); P (recombinase, pCOXS3- phiC31).

The TA_3 _generation of phiC31.22, 31, 34 plant lines were transformed with *Agrobacterium *harboring the pCOXS3-phiC31 vector. Kanamycin resistant transformants that exhibited wild type appearance and growth rate were identified and grown in the greenhouse. Three-week old TR_1 _transformants were tested for the presence of the *phiC31 *gene. PCR amplification by primers ***g ***and ***h ***(Fig. 1b) showed that a majority of the plants harbor the recombinase gene (Fig. 3). The groups of plants that harbor the *phiC31 *gene were designated TR_1_-phiC31.22, 31 and 34 (Table 1).

**Table 1:** PCR analysis of TR_1 _plants

TA Parent line	Plants tested	Positive for recombinase gene ^***a ***^and target locus ^***b***^	Positive for excision ^***c***^	**Positive for excision and negative for unexcised product **^***d***^
phiC31.22	88	65	47	35

phiC31.31	67	31	21	14

phiC31.34	43	19	17	2

^*a *^Primers ***g ***and ***h ***yielded the 0.71 kb *phiC31 *fragment.

^*b *^Primers ***e ***and ***f ***yielded the 1.26 and/or 0.44 kb fragment.

^*c *^Primers ***e ***and ***f ***yielded the 0.44 kb excision fragment.

^*d *^Primers ***e ***and ***f ***failed to detect a 1.26 kb target fragment.

The TR_1_-phiC31 lines were examined using histochemical staining to detect *gusA *encoded β-glucuronidase activity. GUS expression in the TR_1_-phiC31 lines, however, showed variable levels of β-glucuronidase activity. Initially we attributed this reduced activity to lower levels of phiC31-mediated excision, but PCR analysis of lines where GUS activity was weak or undetectable were positive for excision of the target DNA. Given that the screening for GUS activity was not a reliable indicator of phiC31 site-specific recombination, we subsequently utilized PCR to screen for site-specific excision.

With the 65 TR1-phiC31.22, 31 TR_1_-phiC31.31 and 19 TR_1_-phiC31.34 individuals, PCR with primers ***e ***and ***f ***(Fig. 1c) detected a 0.44 kb product expected for site-specific excision (Fig. 3a). However, the 1.26 kb product representing the parental configuration was also detected in some individuals, which indicates the presence of unexcised target DNA. As each individual harbors an independent COXS3-phiC31 T-DNA integration at a different genomic location, with perhaps a different copy number or structural arrangements, the incomplete excision in some individuals may be due to variability in recombinase gene expression.

### Removal of the phiC31 gene by segregation

To determine if the genomic excision event occurred in the germline tissue, we examined whether the excised target was heritably transmitted to the progeny lacking the *phiC31 *gene. This analysis further resolved whether or not the excision reaction was generated *de novo *in each generation. We chose 5 individuals (Table 2) from each of the TR_1_-phiC31.22, TR_1_-phiC31.31 and TR_1_-phiC31.34 families to pollinate wild type recipients. The backcross progenies (BC_1_) were grown without selection and then screened by PCR for the target locus (primers ***e ***and ***f***) and the recombinase gene (primers ***g ***and ***h***), which reveals whether excision occurred (0.44 kb band) or not (1.26 kb band) and if *phiC31 *was present or absent (Fig. 3c, d). With the TR_1_-phiC31.22, TR_1_- phiC31.31 and TR_1_-phiC31.34, 59% (115 of 194), 78% (178 of 227) and 55% (118 of 214) of the BC_1 _plants harbored the target DNA, respectively.

**Table 2:** PCR analysis of BC_1 _and S_1 _plants

TR_**1**_-Parent line	Plants tested	**Positive for target locus **^***a***^	**Positive for excision **^***b***^	**Positive for excision and negative for recombinase gene **^***c***^	**Positive for recombinase gene and negative for target locus **^***d***^
phiC31.22.3	42	23	23	21	0

phiC31.22.15	17	16	16	2	0

phiC31.22.23	68	29	21	3	1

phiC31.22.29	61	44	44	24	1

phiC31.22.87	6	3	3	1	1

					

phiC31.31.1	59	55	28	14	1

phiC31.31.13	44	29	22	10	14

phiC31.31.23	14	8	8	2	4

phiC31.31.29	62	59	59	22	0

phiC31.31.36	48	27	25	13	5

					

phiC31.34.2	43	18	18	1	0

phiC31.34.5	44	23	8	0	2

phiC31.34.9	44	13	13	0	9

phiC31.34.20	42	26	26	0	1

phiC31.34.24	41	38	38	0	0

^*a *^Primers ***e ***and ***f ***yielded the 1.26 and/or 0.44 kb target fragment.

^*b *^Primers ***e ***and ***f ***yielded the 0.44 kb excision fragment.

^*c *^Primers ***e ***and ***f ***yielded the 0.44 kb excision fragment while primers ***g ***and ***h ***failed to detect the 0.71 kb *phiC31 *fragment.

^*d *^Primers ***g ***and ***h ***yielded the 0.71 kb *phiC31 *fragment while primers ***e ***and ***f ***failed to detect the 1.26 and/or 0.44 kb target fragment.

For the five TR_1_-phiC31.22 plants that were backcrossed, 93% of the plants (107 of 115) that harbor the target locus showed excision of the *attB *and *attP*-flanked DNA, with 48% (51 of 107) lacking the recombinase gene (Table 2). Of the TR_1_-phiC31.31 plants, 80% (142 of 178) of target plants showed excision of the *attB *and *attP*-flanked target, and 43% (61 of 142) lack the recombinase gene (Table 2). A total of 87% of the TR_1_- phiC31.34 plants (103 of 118) harbored the target locus with excision of the *attB *and *attP*-flanked DNA, 1% (1 of 103) lacked the recombinase gene (Table 2). The genomic excision 0.44 kb PCR product from two representative individuals from each family was sequenced and examined for conservative recombination. All of the phiC31-mediated excision PCR products sequenced were conservative and site specific (GenBank accession No. GU564447, Fig. 1e).

### BC_1 _progeny for molecular confirmation

**Figure 4:**
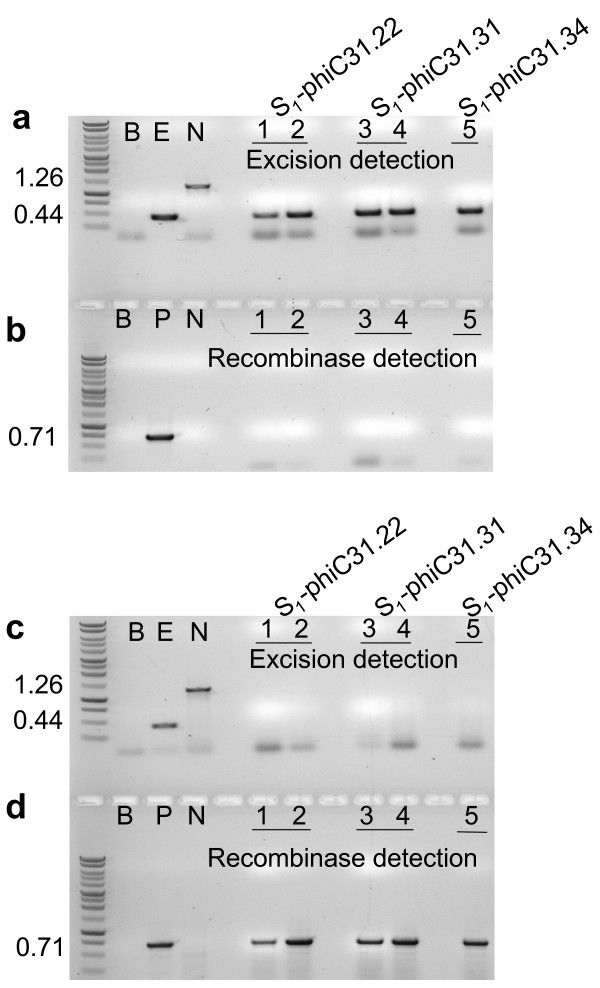
**PCR analysis for site-specific recombination and the presence of the *phiC31 *gene in the S_1 _generation**. PCR reactions (a, c) with primers ***e ***and ***f ***(**Fig. 1**) or (b, d) with primers ***g ***and ***h ***(**Fig. 1**) on representative plant DNAs. a, b) (lanes 1, 2) Self fertilized -Excision only target lines S_1_-phiC31. 22.3.18.1, 22.29.7.1; (lanes 3, 4) S1-phiC31.31.1.1, 31.31.13.1; (lane 5) S_1_-phiC31.34.2.10.1. c, d) Self fertilized - Recombinase only expression lines (lanes 1, 2) S_1_-phiC31.22.3.5.1, 22.15.5.1; (lanes 3, 4) S_1_-phiC31.31.23.10.1, 31.31.36.2; (lane 5) S_1_-phiC31.34.9.2.1. Control lanes are B (blank, no DNA); E (excision, pN3-phiC31_exc_); N (no excision, pN3-phiC31); P (recombinase, pCOXS3-phiC31).

**Figure 5:**
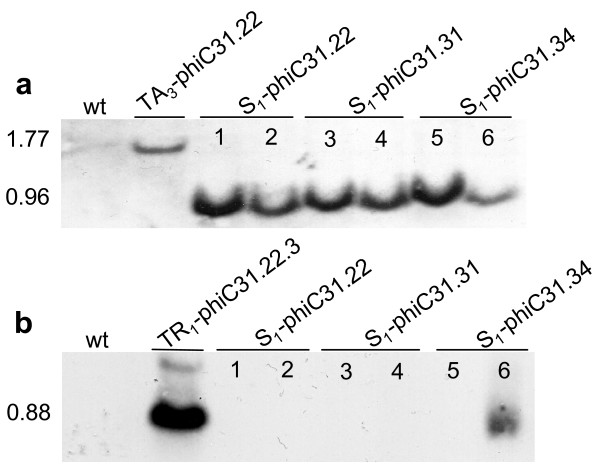
**S_1 _plants examined by Southern blot analysis for excision and segregation of *phiC31 *gene**. a) Genomic DNA cleaved with *Eco*RV hybridized with a ^32^P-labeled GUS1350 probe (**Fig. 1). **b) Genomic DNA digested with *Xho*I and hybridized with a ^32^P-labeled NPT690 probe. Plant lines (lanes 1, 2) S1-phiC31.22.3.18.1, 22.29.7.1; (lanes 3, 4) S_1_- phiC31.31.31.1.1, 31.31.13.1; (lane 5, 6) S_1_-phiC31.34.2.10.1; 34.9.20.1. Control lanes are wt (wild type *Arabidopsis *genomic DNA), TA_3_-phiC31.22, (target lines), TR_1_- phiC31.22.23 (*phiC31 *recombinase expression line).

BC_1 _plants that showed excision but lacked the recombinase gene were self-fertilized to yield progeny designated S_1_-phiC31. PCR analysis on these plants again confirmed excision in the absence of the *phiC31 *recombinase gene (Fig. 4a, b), which indicates germinal transmission of the excision event. For further confirmation, Southern blot hybridization was conducted on some of these S_1 _individuals. The genomic DNA was isolated and cleaved with *Eco*RV, which is expected to liberate either a 1.77 kb or a 0.96 kb fragment from the non-recombined or recombined structure, respectively (Fig. 1a, c). The GUS1350 probe detected the 1.77 kb band in the parental lines but not in the S_1 _plants (Fig. 5a, lanes 1-6). Instead, only the 0.96 kb band was observed for S_1 _plants from the TR_1_-phiC31 lineage. Genomic DNA was also cleaved with *Xho*I, which should liberate a 0.88 kb fragment if the genome were to harbor a COXS3-phiC31 T-DNA. Hybridization with the NPT690 probe detected the *npt*II gene fragment in the parental controls but not in the S_1 _plants determined to be excision positive and *phiC31 *negative (Fig. 5b, lanes 1-5) with the exception of a non-segregated S_1_-phiC31.34.9 plant line that contains both the excision product and the recombinase expression cassette (Fig. 5b, lane 6).

**Figure 6:**
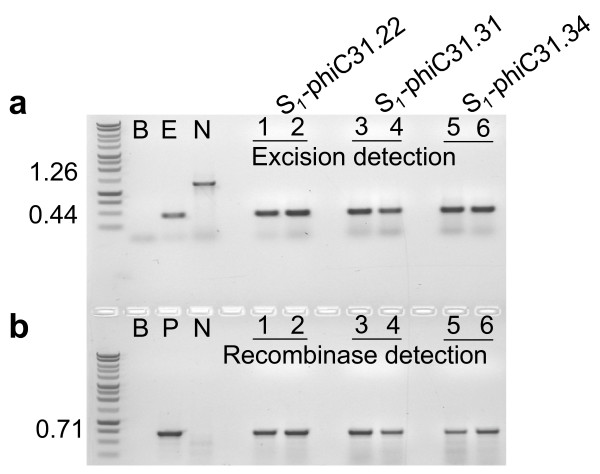
**PCR analysis for site-specific recombination and the presence of the *phiC31 *gene in the MC_1 _generation**. a) PCR reactions with primers ***e ***and ***f ***(**Fig. 1**) or b) with primers ***g ***and ***h ***(**Fig. 1**) on manually crossed lines; (lanes 1, 2) MC_1_-phiC31.22.3, 22.15; (lanes 3, 4) MC_1_-phiC31.31.40, 31.83; (lane 5, 6) MC_1_-phiC31.34.9, 34.20. Control lanes are B (blank, no DNA); E (excision, pN3-phiC31_exc_); N (no excision, pN3-phiC31); P (recombinase, pCOXS3-phiC31).

We further isolated, by segregation, *phiC31 *recombinase expression lines for the purpose of crossing to the original TA_3 _target lines to determine if a genomic excision event could be facilitated using this alternative approach (Fig. 2). Two independent lines from each of the three (TR_1_-phiC31.22, 31 and 34) secondary transformation events were isolated via PCR and designated lines S_1_-COXS3-22.3, 22.15; S_1_-COXS3-31.40, 31.83 and S_1_-COXS3-34.9, 34.20. Each of these six independently isolated lines has been crossed to the original target line TA_3_-phiC31.22. As the lines S_1_-COXS3-22.3 and S_1_-COXS3-22.15 were derived from the original TR_1_-phiC31.22 secondary transformation; crossing them back to the TA_3_-phiC31.22 target line was performed as a positive control for this line of investigation. The manually crossed progenies (MC_1_) were grown without selection and screened by PCR for the target locus (primers ***e ***and ***f***) and the recombinase gene (primers ***g ***and ***h***; Fig. 6). Of the MC_1_-phiC31.22 plants that carried both the target locus and *phiC31 *gene, 61% (19 of 31) of the tested individuals displayed the 0.44 kb excision band in the absence of the unexcised 1.26 kb target band when screened using PCR (Table 3; Fig. 6, lanes 1, 2). Of the MC_1_-phiC31.31 plants with both the target and *phiC31*, 100% (25 of 25) generated only the 0.44 kb excision PCR product (Table 3; Fig. 6, lanes 3, 4). While 92% (34 of 37) of the MC_1_-phiC31.34 individuals generated only the 0.44 kb PCR product derived from an excised genomic target (Table 3; Fig. 6, lanes 5, 6).

**Table 3:** PCR analysis of MC_1 _plants

MC_**1**_-Parent line	Plants tested	**Positive for target locus **^***a***^	**Positive for recombinase gene **^***b***^	**Positive for excision and recombinase gene **^***c***^	**Positive for excision and negative for unexcised product **^***d***^
phiC31.22.3	11	10	11	8	8

phiC31.22.15	34	31	32	23	11

					

phiC31.31.40	17	16	16	8	8

phiC31.31.83	17	17	17	17	17

					

phiC31.34.9	68	44	23	15	15

phiC31.34.20	44	33	40	22	19

^*a *^Primers ***e ***and ***f ***yielded the 1.26 and/or 0.44 kb target fragment.

^*b *^Primers ***g ***and ***h ***failed to detect the 0.71 kb *phiC31 *fragment.

^*c *^Primers ***e ***and ***f ***yielded the 0.44 kb excision fragment and primers ***g ***and ***h ***detect the 0.71 kb *phiC31 *fragment.

^*d *^Primers ***e ***and ***f ***yielded the 0.44 kb excision fragment and failed to detect a 1.26 kb target fragment.

## Discussion

Our interest in site-specific recombination lies in its ability to facilitate crop improvement through controlled engineering of the plant genome. Recently transgenic corn has been deregulated for the production of high lysine, a consumer directed product [22,23]. Further, this transgenic crop was engineered with the assistance of the site-specific recombinase technology for marker removal. Deregulation in this case required extensive studies to ensure that the recombinase mediated excision event was heritably transmitted to subsequent generations in the absence of the recombinase gene [23]. Such agricultural requirements, while obviously necessary, have elicited few detailed studies on the transmission of recombined chromosome transmission to progeny plants. The recombinase systems Cre/*lox*, Flp/*FRT*, R/*RS*, β/*six *and ParA/*MRS *have all been shown capable of germinal transmission *in planta *[19,24-30]. Therefore, our research investigated the publicly available phiC31 recombination system as a potential tool for the precise removal of plant transgenes. In order to demonstrate its utility for crop genome engineering and increase public acceptance of transgenic technology, the potential for predefined nuclear excision events and their germinal transmission was investigated. An advantage of phiC31 over existing recombinase systems is its unidirectional recombination activity, which prevents the re-insertion of the excision product into the genome. In addition, phiC31 has the ability to site-specifically integrate DNA into the host genome [8,13] making this a versatile enzyme.

Our strategy began with the assumption that we could use *gusA *expression as a reporter for site-specific recombination. The pattern of GUS enzyme activity would reveal genomic excision of the target sequence and any tissue specificity in recombination. This strategy, however, failed to perform as expected with initial excised plants being either weak or completely devoid of GUS activity. Subsequent analysis of the original TR_1_-phiC31 progeny confirmed that use of reporter enzyme activity was an unreliable indicator of excision. We had also observed this phenomenon with other constructs used in both *Arabidopsis *and *S. pombe *[8,19]. It is possible that the 54 bp *attB/P *hybrid sequence present within the transcript leader sequence of the *gusA *gene may cause poor expression due to methylation or by some other mechanism that inhibits gene expression. Due to this circumstance, the analysis and scoring of site-specific excision was performed using PCR.

Site-specific excision was detected in all TR_1_-phiC31.22, TR_1_-phiC31.31 and TR_1_-phiC31.34 plants. The majority (72%) of the TR_1_-phiC31.22 and TR_1_-phiC31.31 plants that demonstrated the presence of the excision product did not yield the PCR amplified unexcised target band. This indicates that the phiC31-mediated genomic excision reaction was complete, or nearly so, within many of these TR_1 _plants. The exception was line TR_1_-phiC31.34. Only 12% of the TR_1_-phiC31.34 plants were positive for the 0.44 kb excision band in the absence of the 1.26 kb unexcised target band. This may be due to unfavorable placement of the target construct within the *Arabidopsis *genome. Indeed, although the TR-phiC31.34 lines generated lower levels of recombinase-mediated excision than either the TR-phiC31.22 or TR-phiC31.31 lines, when segregants (derived from TR-phiC31.34) containing only the *phiC31 *expression cassette were manually crossed with TA-phiC31.22 target plants, 92% of the progeny generated only the 0.44 kb excised target PCR product. This indicates that *phiC31 *functions well in these plants, despite performing less efficiently on the TA-phiC31.34 target. The simplest explanation is that the TA-phiC31.34 genomic location or structure was unfavorable to recombination in the germinal tissue.

From analysis of the BC_1 _plants, 85.6% (352 of 411) of those derived from the three TR_1_-phiC31 lines showed excision, while in a previous line of research 77.3% and 99.6% of the BC_1 _plants of the TR_1_-ParA and TR_1_-Cre lines exhibited excision, respectively [19]. By this measure, it appears that the phiC31 recombinase mediated excision efficiency is more effective than ParA and approaching that of the Cre-*lox *system. Although, the majority of the BC_1 _lines displayed excised genomic target, it is difficult to give a precise quantitative assessment of the phiC31 activity since only a modest number of different target locations were thoroughly characterized. Variability in copy number and chromosome locations of the *phiC31 *gene can affect the amount of recombinase protein produced and thus impact the efficiency of the excision reaction observed, making a direct comparison difficult. Other excision strategies for the phiC31 recombinase are being investigated. These include the use of inducible or tissue specific promoters for controllable expression [31] use of self-deleting designs [32] and use of viral inoculation or *Agrobacterium*-infiltration for immediate but transient expression [33,34].

As an alternative method of recombinase introduction into the plant target lines, our lab tested hand pollination between *phiC31 *recombinase expressing plants and pN3-phiC31 target plants. PCR analysis of the manually crossed MC_1 _progeny demonstrated that this is a viable method for the generation of individuals with genomic target excision (Fig. 6). However, it was observed that like secondary *Agrobacterium *transformation with the recombinase expression cassette, the genomic excision results varied between lines (Table 3). Use of a demonstrated recombinase expression line such as phiC31.31.83 (Table 3) enabled sufficient recombinase mediated excision events to fully excise all target DNA when crossed together. It was also observed that segregation of the secondary *Agrobacterium *transformed TR_1 _lines, without benefit of backcrossing, produced excised target and recombinase expression-only T-DNA lines in the TR_2 _and TR_3 _generations (data not shown). This indicates that the *phiC31 *expression T-DNA in these lines was at a single locus or a low number of loci within the genome and that expression was sufficient to facilitate recombination allowing segregation by self- pollination.

Since PCR assays of genomic DNA from leaf tissue only indicates that excision has occurred in somatic cells, we utilized Southern blot analysis to ascertain whether target sequence removal had occurred in the germline. As long as *phiC31 *DNA was present in the genome, or the phiC31 protein was present in the germline cells, the possibility that recombination was generated *de novo *could not be ruled out. Hence, BC_1 _plants were screened by PCR for the absence of the *phiC31 *recombinase gene, and the following generation (S_1 _plants) was confirmed by Southern blot hybridization. As is clearly shown in Fig. 5 lanes #1 - 5, germinal transmission of the genomic excision event in the absence of the *phiC31 *recombinase gene occurred, illustrating that the production of stable lines with the unwanted DNA removed can be achieved.

**Figure 7:**
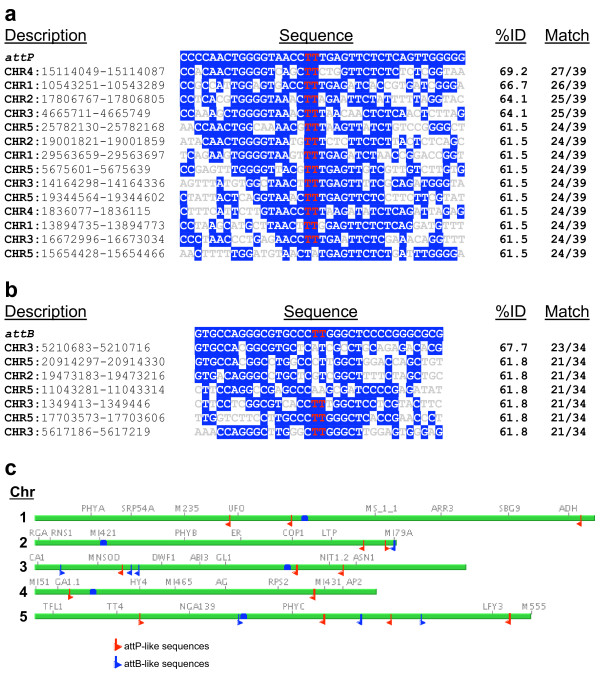
***Arabidopsis *genomic DNA sequences with >60% similarity to phiC31 *attP *and *attB *sites**. a) Alignment of the 39 bp *attP *site with 14 sequences from the *Arabidopsis *genome. b) Alignment of the 34 bp *attB *site with seven sequences from the *Arabidopsis *genome. Nucleotides identical to the *att *site are highlighted with white text and blue backshading. A conserved core domain is highlighted in red text. The chromosomal location coordinates of each sequence are shown on the left, the percent identity and nucleotide match is shown on the right. c) The position and orientation of the 21 *att*-like sequences are displayed on a diagram of the five *Arabidopsis *chromosomes.

Controlled targeted integration with recombinase technology allows the application of more sophisticated recombinase strategies [35]. This technology enables the production of precisely engineered transgenic plants through genome specific transgene integration and has been reported to function in *Arabidopsis*, tobacco and rice [5,36-44] with Cre, Flp and R recombinase systems. The phiC31 recombinase with its uni-directional catalytic activity presents a novel way to facilitate stable site-specific integration events without the elaborate strategies required by the bi-directional systems. Peer-reviewed literature reported that phiC31 is capable of mammalian genome targeting [45,46] and targeted integration into the plastid genome of tobacco [13]. Utilization of phiC31 for genome modification has been facilitated in mammalian species through the identification of cryptic *attB *or *attP *sites as potential locations for transgene introduction [46]. To this end our lab investigated, *in silico*, the presence of sequences similar to the phiC31 *att *sites within the *Arabidopsis thaliana *genome. We used a BLASTn search to investigate whether the *Arabidopsis *genome contains sequences similar to the minimal 34 bp *attB *and 39 bp *attP *sites [12]. The genomic sequences with the highest similarity to the *att *sites exhibited >60% overall nucleotide identity. A total of seven sequences had 21-23 (61.8-67.7%) of the 34 nucleotides conserved with the minimal *attB *sequence, while 14 native sequences had 24-27 (61.5-69.2%) nucleotides in common with the 39 bp *attP *sequence (Fig. 7). While most of the sequences including the best matches for *attP *did contain the conserved core domain presumably essential for phiC31-mediated recombination, only three of the *attB*-like sequences contained the core sequence (Fig. 1d; Fig. 7). It is possible that some of these *att*-like sequences could potentially be used as a native target site for phiC31 mediated integration in *Arabidopsis*. Pseudo phiC31 *attP *sequences in the mouse, bovine and human genomes have been reported and some of them have been shown suitable for integration of introduced DNA [47-49].

Although unlikely, the potential for genomic excision, inversion and translocation mediated by these cryptic *att *sequences in *Arabidopsis *is possible. For excision, *Arabidopsis *chromosomes 3 and 5 carry both *attB *and *attP*-like sequences in direct orientation (Fig. 7). The closest correctly oriented sites are located >500 kb apart on chromosome 3, but the cryptic *attB *does not contain a conserved core domain. Although it is theoretically possible that genomic recombination could occur via endogenous *att*-like sequences, the *OXS3 *promoter-*phiC31 *plants did not exhibit compromised viability, morphological or growth defects. This differs from earlier observations using a *35S-phiC31 *construct where *Arabidopsis *plants with crinkled leaves were common [C. Day and D.W. Ow, unpublished data]. Hence, this underscores the importance in controlling expression of the recombinase gene through appropriate use of promoters.

## Conclusion

The purpose of the research was to provide proof-of-concept that the phiC31 recombinase can mediate site-specific genome modification in the plant germline tissue without affecting fecundity. The research established that the excision event was passed to subsequent generations in the absence of phiC31 and that the excision of *attB *and *attP*-flanked DNA from the plant genome was a conservative site-specific event. In a majority of the phiC31 lines examined (11 out of 15), at least one BC_1 _segregant was recovered that contained a germinally transmitted excision event lacking the *phiC31 *gene. These results were validated with Southern blot hybridization and demonstrate that the secondary transformation strategy used in this study is feasible for the production of marker-free transgenic plants. This approach may prove particularly useful in those species where cross pollination is not possible or undesirable. We further demonstrate that an alternative approach to marker removal where the recombinase is introduced into the excision test target plants with cross pollination is also a viable strategy. Molecular analysis confirmed that the genomic excision was site-specific and conservative. Therefore, taken together the results clearly establish that the phiC31 system performs genomic excision, generating stable transgenic recombinase-free *Arabidopsis *plants with unwanted DNA removed.

## Methods

### DNA constructs

pN3-phiC31 (GenBank accession No. GU564446), (Fig. 1a): An *Nhe*I-*attB*-stuffer-*attP- Asc*I fragment was retrieved from pPB-phiC31 [8] and inserted into binary vector pCambia-1301 http://www.cambia.org/daisy/cambia in which the *Nco*I site between *35S *and *gusA *had been changed to *Spe*I and *Asc*I. The vector contains *hpt*II (*hygromycin phosphotransferase *II) for selection in plants outside the region of site-specific excision to allow for progeny tracking. The pN3-phiC31_exc _vector for control lanes (Fig. 3, 4 and 6, lane E) was generated by removal of the non-coding stuffer region by recombinase-mediated excision in bacteria.

pCOXS3-phiC31 (GenBank accession No. GU564445), (Fig. 1b): The *phiC31 *ORF was Phusion (NEB, New England Biolabs) PCR amplified with a 5' *Asc*I and 3' *Spe*I sites (underlined) and inserted into pCOXS3-ParA [19] to generate the final construct. Primers used were 5'-AGTCGGCGCGCCATGACACAAGGGGTTGTGAC-3' and 5'-AGTCACTAGTCTACGCCGCTACGTCTTC-3'. The 1.5 kb fragment promoter of the *OXS3 *gene (AGI At5g56550) from *Arabidopsis thaliana *(ecotype: L*er*) was used to express the *phiC31 *ORF, as previously described [19,20]. The pCAMBIA 2300 http://www.cambia.org/daisy/cambia, binary vector with *npt*II (*neomycin phosphotransferase *II) for plant selection was used as the backbone for plant transformation.

*Agrobacterium tumefaciens *GV3101 was used for transformation of *Arabidopsis *(ecotype: L*er*) by the floral dip method [50] modified by adding 0.01% Silwet L-77 (Lehle Seeds, Round Rock, TX) to the infiltration medium. Primary transformants were selected on 1× MS medium (Sigma), 1% sucrose, 0.7% agar with 20 μg/ml hygromycin or 50 μg/ml kanamycin as needed for 10 days prior to cultivation in soil.

### PCR analysis

Genomic DNA was extracted by grinding a single leaf in 400 μl of buffer (200 mM Tris HCl pH 7.8, 250 mM NaCl, 25 mM EDTA, 0.5% SDS). After centrifugation, the isopropanol precipitated pellet was washed with 70% ethanol and resuspended in 50 μl of H_2_O. Two μl of genomic DNA in 25 μl volume was used per PCR reaction. Primers were (Fig. 1): ***e ***(5'-ATATCTCCACTGACGTAAGG-3'), ***f ***(5'-ATCATCATCATAGACACACG-3' for N3-phiC31); ***g ***(5'-AGTCGGCGCGCCATGACACAAGGGGTTGTGAC-3'), ***h ***(5'- GTGCGTCTTGATCTCACG-3' for *phiC31*). Gel images were digitized with a resolution of 200 dpi in black on white background TIF format.

### Southern blot analysis

Genomic DNA was extracted from plant aerial portions using a modified cetyl- trimethyl-ammonium bromide method as described [51]. The 0.79 kb GUS1350 and 0.69 kb NPT690 ^32^P-labeled probes were produced by *Taq*™ polymerase (Promega) using primers 5'-CAAGACCCTTCCTCTATATAAG-3' and 5'-CGAGTTCATAGAGATAACCTTC-3' for GUS1350 and primers 5'- GATTGAACAAGATGGATTGCACGC-3' and 5'- CCACAGTCGATGAATCCAGAAAAGC-3' for NPT690.

## Authors' contributions

JT designed the approach, constructed the plasmids, collected data, interpreted the research results, drafted and edited the manuscript. JT supervised RC, prepared and submitted the manuscript. RC provided technical assistance with plant maintenance, DNA preparation, PCR data collection and analysis. Participated with manuscript preparation and editing. RT provided bioinformatics research on the cryptic *attB *and *attP *sites and performed Southern blot hybridization and analysis. Participated in the drafting and editing of the manuscript. YY provided assistance on background studies, data interpretation and manuscript editing. DO provided data interpretation and manuscript editing. All authors have read and approved the final manuscript.

## References

[B1] Herring RJ Opposition to transgenic technologies: ideology, interests and collective action frames Nat Rev Genet 2008 9 458 63 10.1038/nrg2338 18487989

[B2] Cellini F Chesson A Colquhoun I Constable A Davies HV Engel KH Gatehouse AM Karenlampi S Kok EJ Leguay JJ Lehesranta S Noteborn HP Pedersen J Smith M Unintended effects and their detection in genetically modified crops Food Chem Toxicol 2004 42 1089 1125 10.1016/j.fct.2004.02.003 15123383

[B3] Hare PD Chua NH Excision of selectable marker genes from transgenic plants Nat Biotechnol 2002 20 575 80 10.1038/nbt0602-575 12042860

[B4] Hohn B Levy AA Puchta H Elimination of selection markers from transgenic plants Curr Opin Biotechnol 2001 12 139 143 10.1016/S0958-1669(00)00188-9 11287227

[B5] Chawla R Ariza-Nieto M Wilson AJ Moore SK Srivastava V Transgene expression produced by biolistic-mediated, site-specific gene integration is consistently inherited by the subsequent generations Plant Biotechnol J 2006 4 209 18 10.1111/j.1467-7652.2005.00173.x 17177797

[B6] Sauer B Functional expression of the cre-lox site-specific recombination system in the yeast Saccharomyces cerevisiae Mol Cell Biol 1987 7 6 2087 2096 3037344 10.1128/mcb.7.6.2087-2096.1987 PMC365329

[B7] Miki B McHugh S Selectable marker genes in transgenic plants: applications, alternatives and biosafety J Biotechnology 2004 107 3 193 232 10.1016/j.jbiotec.2003.10.011 14736458

[B8] Thomson JG Ow DW Site-specific recombination systems for the genetic manipulation of eukaryotic genomes Genesis 2006 44 465 476 10.1002/dvg.20237 16981199

[B9] Keravala A Groth AC Jarrahian S Thyagarajan B Hoyt JJ Kirby PJ Calos MP A diversity of serine phage integrases mediate site-specific recombination in mammalian cells Mol Genet Genomics 2006 276 2 135 146 10.1007/s00438-006-0129-5 16699779

[B10] Thomason LC Calendar R Ow DW Gene insertion and replacement in *Schizosaccharomyces pombe *mediated by the *Streptomyces *bacteriophage phiC31 site-specific recombination system Mol Genet Genomics 2001 265 1031 1038 10.1007/s004380100498 11523775

[B11] Thorpe HM Smith MC *In vitro *site-specific integration of bacteriophage DNA catalyzed by a recombinase of the resolvase/invertase family Proc Natl Acad Sci USA 1998 95 10 5505 5510 10.1073/pnas.95.10.5505 9576912 PMC20407

[B12] Groth AC Olivares EC Thyagarajan B Calos MP A phage integrase directs efficient site-specific integration in human cells Proc Natl Acad Sci USA 2000 97 11 5995 6000 10.1073/pnas.090527097 10801973 PMC18547

[B13] Lutz K Corneille S Azhagiri AK Svab Z Maliga P A novel approach to plastid transformation utilizes the phiC31 phage integrase Plant J 2004 37 906 913 10.1111/j.1365-313X.2004.02015.x 14996222

[B14] Kittiwongwattana C Lutz K Clark M Maliga P Plastid marker gene excision by the phiC31 phage site-specific recombinase Plant Mol Biol 2007 64 137 43 10.1007/s11103-007-9140-4 17294253

[B15] Lutz KA Maliga P Plastid genomes in a regenerating tobacco shoot derive from a small number of copies selected through a stochastic process Plant J 2008 56 975 83 10.1111/j.1365-313X.2008.03655.x 18702667

[B16] Gils M Marillonnet S Werner S Grützner R Giritch A Engler C Schachschneider R Klimyuk V Gleba Y A novel hybrid seed system for plants Plant Biotechnol J 2008 6 3 226 235 10.1111/j.1467-7652.2007.00318.x 18086236

[B17] Rubtsova M Kempe K Gils A Ismagul A Weyen J Gils M Expression of active Streptomyces phage phiC31 integrase in transgenic wheat plants Plant Cell Rep 2008 27 1821 1831 10.1007/s00299-008-0604-z 18797873

[B18] Bayley CC Morgan M Dale EC Ow DW Exchange of gene activity in transgenic plants catalyzed by the Cre-*lox *site-specific recombination system Plant Mol Biol 1992 18 353 361 10.1007/BF00034962 1310059

[B19] Thomson JG Yau YY Blanvillain R Chiniquy D Thilmony R Ow DW ParA resolvase catalyzes site-specific excision of DNA from the *Arabidopsis *genome Transgenic Res 2009 18 2 237 48 10.1007/s11248-008-9213-4 18704739

[B20] Blanvillain R Kim JH Lima A Ow DW OXIDATIVE STRESS 3 is a chromatin-associated factor involved in tolerance to heavy metals and oxidative stress Plant J 2009 57 4 654 665 10.1111/j.1365-313X.2008.03717.x 18980652

[B21] Laubinger S Zeller G Henz SR Sachsenberg T Widmer CK Naouar N Vuylsteke M Schölkopf B Rätsch G Weigel D At-TAX: a whole genome tiling array resource for developmental expression analysis and transcript identification in *Arabidopsis thaliana* Genome Biol 2008 9 R112 10.1186/gb-2008-9-7-r112 18613972 PMC2530869

[B22] Ow DW GM maize from site-specific recombination technology, what next? Curr Opin Biotechnol 2007 18 115 120 10.1016/j.copbio.2007.02.004 17353124

[B23] Environmental USDA-APHIS: Assessment of Petition 04-229-01P http://www.aphis.usda.gov/brs/aphisdocs/04_22901p_pea.pdf

[B24] Dale EC Ow DW Gene transfer with subsequent removal of the selection gene from the host genome Proc Natl Acad Sci USA 1991 88 10558 10562 10.1073/pnas.88.23.10558 1660141 PMC52968

[B25] Russell SH Hoopes JL Odell JT Directed excision of a transgene from the plant genome Mol Gen Genet 1992 234 49 59 1495484 10.1007/BF00272344

[B26] Srivastava V Ow DW Single-copy primary transformants of maize obtained through the co-introduction of a recombinase-expressing construct Plant Mol Biol 2001 46 561 566 10.1023/A:1010646100261 11516149

[B27] Lyznik LA Rao KV Hodges TK FLP-mediated recombination of FRT sites in the maize genome Nucleic Acids Res 1996 24 3784 3789 10.1093/nar/24.19.3784 8871559 PMC146161

[B28] Hu Q Kononowicz-Hodges H Nelson-Vasilchik K Viola D Zeng P Liu H Kausch AP Chandlee JM Hodges TK Luo H FLP recombinase-mediated site-specific recombination in rice Plant Biotechnol J 2008 6 176 188 10.1111/j.1467-7652.2007.00310.x 18021190

[B29] Grønlund JT Stemmer C Lichota J Merkle T Grasser KD Functionality of the β/six Site-Specific Recombination System in Tobacco and Arabidopsis: A Novel Tool for Genetic Engineering of Plant Genomes Plant Mol Biol 2007 63 545 556 10.1007/s11103-006-9108-9 17131098

[B30] Nanto K Ebinuma H Marker-free site-specific integration plants Transgenic Res 2008 17 337 344 10.1007/s11248-007-9106-y 17588210

[B31] Zuo J Niu QW Chua NH Technical advance: An estrogen receptor-based transactivator XVE mediates highly inducible gene expression in transgenic plants Plant J 2000 24 265 273 10.1046/j.1365-313x.2000.00868.x 11069700

[B32] Hoff T Schnorr KM Mundy J A recombinase-mediated transcriptional induction system in transgenic plants Plant Mol Biol 2001 45 41 49 10.1023/A:1006402308365 11247605

[B33] Jia H Pang Y Chen X Fang R Removal of the selectable marker gene from transgenic tobacco plants by expression of Cre recombinase from a tobacco mosaic virus vector through agroinfection Transgenic Res 2006 15 375 384 10.1007/s11248-006-0011-6 16779652

[B34] Kopertekh L Schiemann J Agroinfiltration as a tool for transient expression of cre recombinase in vivo Transgenic Res 2005 14 793 798 10.1007/s11248-005-8293-7 16245170

[B35] Ow DW Litz RE, Scorza R Site-Specific Recombination for Plant Genetic Engineering: Strategy for Agro-Mediated Gene Stacking ISHS Acta Horticulturae 738, International Symposium on Biotechnology of Temperate Fruit Crops and Tropical Species: Mar31, 2007; The Netherlands, Drukkerij Jansen BV 2007 117 127

[B36] Albert H Dale EC Lee E Ow DW Site-specific integration of DNA into wild-type and mutant *lox *sites placed in the plant genome Plant J 1995 7 649 659 10.1046/j.1365-313X.1995.7040649.x 7742860

[B37] Choi S Begum D Koshinsky H Ow DW Wing RA A new approach for the identification and cloning of genes: the pBACwich system using Cre/lox site-specific recombination Nucleic Acids Res 2000 28 E19 10.1093/nar/28.7.e19 10710436 PMC102802

[B38] Day CD Lee E Kobayashi J Holappa LD Albert H Ow DW Transgene integration into the same chromosome location can produce alleles that express at a predictable level, or alleles that are differentially silenced Genes Dev 2000 14 2869 2880 10.1101/gad.849600 11090134 PMC317066

[B39] Nanto K Yamada-Watanabe K Ebinuma H *Agrobacterium *-mediated RMCE approach for gene replacement Plant Biotechnol J 2005 3 203 214 10.1111/j.1467-7652.2005.00118.x 17173620

[B40] Nanto K Ebinuma H Marker-free site-specific integration plants Transgenic Res 2008 17 337 344 10.1007/s11248-007-9106-y 17588210

[B41] Srivastava V Ow DW Biolistic mediated site-specific integration in rice Molecular Breeding 2002 8 345 350 10.1023/A:1015229015022

[B42] Srivastava V Ariza-Nieto M Wilson AJ Cre-mediated site-specific gene integration for consistent transgene expression in rice Plant Biotech J 2004 2 169 179 10.1111/j.1467-7652.2003.00061.x 17147608

[B43] Vergunst AC Hooykaas PJ Cre/lox-mediated site-specific integration of *Agrobacterium *T-DNA in *Arabidopsis *thaliana by transient expression of cre Plant Mol Biol 1998 38 393 406 10.1023/A:1006024500008 9747847

[B44] Vergunst AC Jansen LE Hooykaas PJ Site-specific integration of *Agrobacterium *T-DNA in *Arabidopsis thaliana *mediated by Cre recombinase Nucleic Acids Res 1998 26 2729 2734 10.1093/nar/26.11.2729 9592161 PMC147585

[B45] Belteki G Gertsenstein M Ow DW Nagy A Site-specific cassette exchange and germline transmission with mouse ES cells expressing phiC31 integrase Nat Biotechnol 2003 21 321 324 10.1038/nbt787 12563279

[B46] Thyagarajan B Olivares EC Hollis RP Ginsburg DS Calos MP Site-specific genomic integration in mammalian cells mediated by phage phiC31 integrase Mol Cell Biol 2001 21 12 3926 3934 10.1128/MCB.21.12.3926-3934.2001 11359900 PMC87055

[B47] Thyagarajan B Guimarães MJ Groth AC Calos MP Mammalian genomes contain active recombinase recognition sites Gene 2000 244 1-2 47 54 10.1016/S0378-1119(00)00008-1 10689186

[B48] Chalberg TW Portlock JL Olivarer EC Thyagarajan B Kirby PJ Hillman RT Hoelters J Calos MP Integration specificity of phage phiC31 integrase in the human genome J Mol Bio 2006 357 28 48 10.1016/j.jmb.2005.11.098 16414067

[B49] Ou HL Huang Y Qu LJ Xu M Yan JB Ren ZR Huang SZ Zeng YT A phiC31 integrase-mediated hotspot in favor of transgene expression exists in bovine genome FEBS J 2009 276 155 63 10.1111/j.1742-4658.2008.06762.x 19019083

[B50] Clough SJ Bent AF Floral dip: a simplified method for *Agrobacterium *- mediated transformation of *Arabidopsis thaliana* Plant J 1998 16 735 743 10.1046/j.1365-313x.1998.00343.x 10069079

[B51] Sambrook J Russell DW Argentine J Southern Blot Hybridization Molecular Cloning A Laboratory Manual 2001 3 NewYork: Cold Spring Harbor Press 6.33 6.46

